# Uncovering the role of ferroptosis in Bietti crystalline dystrophy and potential therapeutic strategies

**DOI:** 10.1186/s12964-024-01710-x

**Published:** 2024-07-11

**Authors:** Chang Shen, Qianjie Yang, Kuangqi Chen, Huiling Ma, Xiawei Wang, Jianping Tong, Ye Shen, Hongguang Cui

**Affiliations:** Department of Ophthalmology, The First Affiliated Hospital, Zhejiang University School of Medicine, Hangzhou, China

**Keywords:** Bietti crystalline dystrophy, Ferroptosis, Polyunsaturated fatty acids, NCOA4, Deferiprone

## Abstract

**Purpose:**

Bietti crystalline dystrophy (BCD) is an inherited retinal degeneration disease caused by mutations in the *CYP4V2* gene. Currently, there is no clinical therapy approach available for BCD patients. Previous research has suggested that polyunsaturated fatty acids (PUFAs) may play a significant role in the development of BCD, implicating the involvement of ferroptosis in disease pathogenesis. In this work, we aimed to investigate the interplay between ferroptosis and BCD and to detect potential therapeutic strategies for the disease.

**Methods:**

Genetic-edited RPE cell line was first established in this study by CRISPR-Cas9 technology. *Cyp4v3* (the homologous gene of human *CYP4V2)* knock out (KO) mice have also been used. Lipid profiling and transcriptome analysis of retinal pigment epithelium (RPE) cells from *Cyp4v3* KO mice have been conducted. Ferroptosis phenotypes have been first investigated in BCD models in vitro and in vivo, including lipid peroxidation, mitochondrial changes, elevated levels of reactive oxygen species (ROS), and altered gene expression. Additionally, an iron chelator, deferiprone (DFP), has been tested in vitro and in vivo to determine its efficacy in suppressing ferroptosis and restoring the BCD phenotype.

**Results:**

*Cyp4v3* KO mice exhibited progressive retinal degeneration and lipid accumulation, similar to the BCD phenotype, which was exacerbated by a high-fat diet (HFD). Increased levels of PUFAs, such as EPA (C22:5) and AA (C20:4), were observed in the RPE of *Cyp4v3* KO mice. Transcriptome analysis of RPE in *Cyp4v3* KO mice revealed changes in genes involved in iron homeostasis, particularly an upregulation of NCOA4, which was confirmed by immunofluorescence. Ferroptosis-related characteristics, including mitochondrial defects, lipid peroxidation, ROS accumulation, and upregulation of related genes, were detected in the RPE both in vitro and in vivo. Abnormal accumulation of ferrous iron was also detected. DFP, an iron chelator administration suppressed ferroptosis phenotype in *CYP4V2* mutated RPE. Oral administration of DFP also restored the retinal function and morphology in *Cyp4v3* KO mice.

**Conclusion:**

This study represented the first evidence of the substantial role of ferroptosis in the development of BCD. PUFAs resulting from *CYP4V2* mutation may serve as substrates for ferroptosis, potentially working in conjunction with NCOA4-regulated iron accumulation, ultimately leading to RPE degeneration. DFP administration, which chelates iron, has demonstrated its ability to reverse BCD phenotype both in vitro and in vivo, suggesting a promising therapeutic approach in the future.

**Supplementary Information:**

The online version contains supplementary material available at 10.1186/s12964-024-01710-x.

## Introduction

Bietti crystalline dystrophy (BCD), which was first reported by Gian Battista Bietti in 1937 [1], is an inherited retinal degeneration disease. As an autosomal recessive inherited disease, the penetration frequency of the pathogenic allele of BCD is 1:67000 worldwide [2], which is much more frequent in East Asians as 1:25000, especially in China, with the allele frequency estimated to be 0.5% [3]. Crystalline deposits in the retina and cornea are typical signs of BCD, with developing RPE and retina atrophy [4]. With crystalline deposits appearing, patients suffer from night blindness and vision defects from their 20s and develop into whole blindness in the 50s [4]. Currently, there is no clinical therapeutic approach for these patients.

*CYP4V2*, which encodes protein as the second polypepostnatalptide of cytochrome P450 (CYP) family 4 subfamily V, was identified as the responsible gene for BCD by Li A, et al. in 2000 [5]. CYP family serves as an enzyme in fatty acid metabolism [6], and for *CYP4V2*, saturated and unsaturated fatty acids are its substrate [7]. In the retina, *CYP4V2* is dominantly expressed among the CYP family, and mostly expressed in RPE, which indicates the disease onset [8].

Several researchers have focused on BCD pathogenesis, which all revealed abnormal lipid metabolism in blood serum and lymphocytes of BCD patients [9, 10]. RPE of mice model or iPSC-derived RPE has been used to study the specific lipid metabolism in the eye, which revealed PUFAs accumulation in RPE including C20:4 (arachidonic acid, AA), C22:5 (eicosapentaenoic acid, EPA), C22:6 (docosahexaenoic acid, DHA) [11–13]. Inflammatory response and mitochondria stress have also been detected in these models [12–14]. While, the mechanism by which PUFAs accumulation leads to RPE death remained unclear, posing challenges for the development of therapeutic strategies for the disease.

Ferroptosis, an iron-dependent lipid peroxidation, is a common cell death that exists in a variety of biological processes [15]. Being proposed in 2012 [16], ferroptosis has been detected in several pathologies involving aging, immunity, and neural degeneration [17]. In the retina, ferroptosis also plays an important role in several diseases, such as age-related macular degeneration (AMD) [18], glaucoma [19], optic neuropathy [20], as well as inherited retinal degeneration [21]. Lipids play a crucial role in ferroptosis, while different lipids yield markedly diverse effects. Of them, polyunsaturated fatty acids (PUFAs) promote ferroptosis indirectly [22], by being activated by acylcoenzyme A (CoA) synthetase long-chain family member 4 (ACSL4) and integrated into membrane lipids, thereby facilitating ferroptosis [23].

In BCD, PUFAs accumulate in RPE and lead to RPE cell death [11–14]. RPE cells serve as crucial centers within the retina for lipid metabolism, including the processing of PUFAs [24]. Based on the involvement of PUFAs in ferroptosis and the active lipid metabolism of RPE cells, we hypothesize that ferroptosis may contribute to the pathogenesis of BCD.

To test our hypothesis, genetic-edited cellular and animal models have been established in this research. Phenotypes of ferroptosis including lipid peroxidation, mitochondria changes, ROS elevation, and gene expression changes have been explored. Finally, an iron chelator, DFP, has been used in vitro and in vivo to test its effect on inhibiting ferroptosis and recovering BCD phenotype.

## Methods

### Animals

C57BL/6J mice were used in this study. *Cyp4v3* (the homologous gene of human *CYP4V2*) KO mice constructed on C57BL/6J mice were donated from Professor Wei Li’s lab from State Key Laboratory of Stem Cell and Reproductive Biology, Institute of Zoology, Chinese Academy of Sciences, Beijing, China. Wild type (wt) mice were purchased from Gempharmatech Co., Ltd, Jiangsu, China. All mice were housed in animal facilities of The First Affiliated Hospital, Zhejiang University School of Medicine, Zhejiang, China. The study protocols were approved by the Animal Research Committee of The First Affiliated Hospital, Zhejiang University School of Medicine (Approval number: 1515, 2022). High-fat chow (60% fat, D12492, Research Diets, USA) was used for the high fat diet (HFD). Iron chelator, deferiprone (DFP, HY-B0568, MedChemExpress, Shanghai, China), was soluted at 1 mg/ml and added to mice’s drinking water. The DFP water was changed daily during administration. The *Cyp4v3* KO mice were administered a high fat diet (HFD) with or without a DFP drink from week 3 (Fig. 6. A). The Mice were randomely divided into groups in this study as follow:

GROUP 1, 12-month-old *Cyp4v3* KO mice;

GROUP 2, 12-month-old wt mice;

GROUP 3, 6-month-old *Cyp4v3* KO mice;

GROUP 4, *Cyp4v3* KO mice fed with 1-month HFD, the mice were 7 weeks old at the experiment;

GROUP 5, wt mice fed with 1-month HFD, the mice were 7 weeks old at the experiment;

GROUP 6, *Cyp4v3* KO mice fed with 1-month HFD and DFP, the mice were 7 weeks old at the experiment;

Sample sizes were determined based on similar ophthalmology studies with mice. The mice were randomly assigned to experimental groups, ensuring an equal distribution of gender among the groups. Investigators were not blinded to the group allocation.

### Generation of *CYP4V2* mutated ARPE-19 cells

Adult RPE cell line-19 (ARPE-19) cells were obtained from (Procell Life Science & Technology Co., Ltd., Wuhan, China). *CYP4V2* mutated (mt) ARPE-19 cells were generated by CRISPR-Cas9 technology. The sequence target was designed to exon1 of human *CYP4V2*. Three sgRNAs were designed and sgRNA: GACCAGACTGGCGCCGGCCA was used in further experiments due to the highest knockout efficiency. Gene targeting plasmid encoding sgRNA and Cas9 protein were electroporated into 5 × 10^6^ ARPE-19 cells by Neon Transfection Kit (Thermofisher, America). Cells were seeded into plates 3–4 days after electroporation at the ratio of 1:20. After single clone formation, every clone was separated and seeded into a 48-well plate for further genotype identification. The primers used for Sanger sequencing were: F, TCTTTCGCTTTCGGCTGGGGCG; R, GCTCACTTTGGGATGGGGCACTAGCAGTG. Cells with specific genotypes were expanded and cultured for further use.

### Cell culture

ARPE-19 cells and *CYP4V2* mt ARPE-19 cells were cultured in high-glucose Dulbecco’s modified Eagle’s medium with GlutaMax (DMEM, C11995500BT, Gibco, Carlsbad, CA, USA) with 10% fetal bovine serum (FBS, 10,099,141 C, Gibco, Carlsbad, CA, USA) and 1% penicillin-streptomycin (15,140,148, Gibco, Carlsbad, CA, USA) at 37 °C and 5% CO2. Arachidonic acid and eicosapentaenoic acid (AA, MFCD00004417; EPA, MFCD00065716, Macklin, Shanghai, China) were eluted in 2% fatty acid-free BSA (A2000-1, APPLYGEN, Beijing, China) at concentration of 8mM for storage. Different concentrations of AA and EPA (10µM, 50µM, 100µM) were added to the basic medium of ARPE-19 cells and *CYP4V2*-mt ARPE-19 cells. The medium contained AA or EPA was changed every 2–3 days. DFP was added in basic medium at different concentrations, and removed after 4 h. Cell viability was measured by cell counting kit-8 (HY-K0301, MedChemExpress, Shanghai, China).

### In vivo retinal photography and function evaluation

Ultra-wide field fundus autofluorescence (UWF-AF) imaging was performed by Daytona funduscope (OPTOS, UK) with an excitation wavelength of 488 nm. Before the examination, 1.25% tribromoethanol (0.2 ml/10 g, Aibei, Nanjing, China) was injected intraperitoneal for anesthesia. Pupils were dilated with 0.5% tropicamide drops at least 10 min before examination. After the pupil was dilated, UWF-AF images were captured for both 2 eyes of one mouse.

Retinal function was evaluated by electroretinogram (ERG). ERG was captured by Electrophysiological Diagnostic Systems (Roland, German). Mice were dark-adapted overnight before perforation. The dim red light was used when procedures were performed. The anesthesia procedure was described above. The pupils were dilated with 0.5% tropicamide at least 10 min before examination. The corneal surface was anesthetized and moistened by 0.5% proparacaine eye drops. 3 needle electrodes were inserted in the cheek and buttocks of the mice, while 2 ring electrodes were placed on the cornea of the mice. The scotopic ERG responses were recorded at stimulus intensities of 0.01, 0.1, 1, and 10 cd.s/m^2^ with an interstimulus interval of 5, 10, 10, and 10 s, respectively, between stimulus flashes. Five responses were averaged per result. Amplitudes of a wave and b wave were statistically analyzed.

### Transcriptome profiling and analysis

After 1 month HFD diet, *Cyp4v3* KO mice and control mice were executed by cervical dislocation for transcriptome profiling. Total RNA was treated using the mRNA enrichment method. The raw data were subjected to quality control to determine whether the sequencing data were suitable for subsequent analysis. Then, sequencing was carried out using a DNBSEQ platform (BGI-Shenzhen, China). Analysis was performed on HISAT (Hierarchical Indexing for Spliced Alignment of Transcripts [25].

### Histology and immunofluorescence of Retina

Animals were executed by cervical dislocation. Eyeballs were extracted and harvested in eyeball fixative solution for paraffin sections. Serial paraffin Sect. (7 μm) were used for hematoxylin and eosin (HE) staining or immunofluorescence staining. For HE staining, after dewaxing and hydrating, the sections were stained with HE (BP0211, Baiaosi, Hubei, China) using the standard methods. For immunofluorescence, the sections were incubated with antibodies at 4 °C overnight. Then slices were incubated with secondary antibodies for 1 h at room temperature. After incubating in DAPI (62,248, Thermofisher, USA) for 10 min at room temperature. Antibodies used for staining were 4-HNE (ab48506, 1:200, Abcam, USA), FTH1 (A19544, 1:200, Abclonal, China), NCOA4 (ab314553, 1:200, Abcam, USA), IBA1 (ab107159, 1:200, Abcam, USA). Eyeballs for cryosection were extracted and frozen in liquid nitrogen until section. The frozen retinal sections were stained with Oil Red O solution (BP0340, Baiaosi, Hubei, China) and DHE (D7008-10, Sigma, St. Louis, MO, USA). All images were captured by an Olympus FV3000 (Olympus, Tokyo, Japan) microscope.

### Free fatty acid profile analysis

After a 1-month HFD diet, *Cyp4v3* KO mice and control mice were executed by cervical dislocation for FFA profiling. The entire eyes were meticulously removed from the eye sockets. The RPE was dissected according to a previously published protocol [26]. Subsequent experiments were carried out using an Exion UPLC-QTRAP 6500 PLUS (SCIEX, California, USA) liquid chromatography/mass spectrometer. All analyses were conducted in the electrospray ionization mode under the following conditions: curtain gas = 20, ion spray voltage = 5500 V, temperature = 400 °C, ion source gas 1 = 35, ion source gas 2 = 35. The lipids were extracted from the samples using the improved Bligh/Dyer extraction method (double extraction) with the appropriate internal labeling [27]. A Phenomenex Luna 3 m silica column (inner diameter 150 × 2.0 mm) was utilized to separate various polar lipids under the following conditions: mobile phase A (chloroform: methanol: ammonia = 89.5:10:0.5) and mobile phase B (chloroform: methanol: ammonia: water = 55:39:0.5:5.5). Polar lipids of different types were separated by NP-HPLC. The gradient of mobile phase A was held at 95% for 5 min, then linearly reduced to 60% within 7 min and held for 4 min, then further reduced to 30% and held for 15 min. Finally, the initial gradient was held for 5 min. Multiple reaction monitoring conversion was established for mass spectrometry to enable comparative analysis of various polar lipids [26]. Various polar lipids were quantified by adding internal standards d31-16:0 and d8-20:4.

### Transmission electron microscopy (TEM)

Enucleated eyeballs were immediately put into precooled 2.5% glutaraldehyde. Within the glutaraldehyde solution, the corneas, lens, and vitreous humor were meticulously extracted, while the retinas were individually isolated and preserved in a fresh glutaraldehyde solution for 24 h at 4 °C. Subsequently, the preserved retinas underwent preparation for TEM imaging following established procedures. The mitochondria of the RPE were observed using an HT7650 transmission electron microscope (Hitachi, Tokyo, Japan). Mitochondrial sizes were quantified using Image J software. Pixel size was calibrated as per the image scale, and the freehand selection tool was used to outline mitochondrial regions. Area measurements were then performed.

### SiRNA interference

To silence gene expression, 20 nM siRNA was transfected into the indicated cells using standard procedures with jetPRIME® (Polyplus) according to the manufacturer’s instructions. Cells were stimulated and harvested for RNA or cell extracts for further analysis 48 h after transfection. The following siRNA sequences were used: GGGCUGAACAGCAAAUUAATT.

### MDA and GSH measurement

Malondialdehyde (MDA) and glutathione (GSH) serve as important biochemical markers that signify the levels of oxidative stress present. The MDA levels in the RPE were determined utilizing an MDA Assay Kit (A003-1-2; Jiancheng, Jiangsu, China), while the GSH levels were measured with a GSH and GSSG Assay Kit (S0053; Beyotime, Shanghai, China). Subsequently, the measured values were adjusted based on the protein concentration of the samples.

### Cell immunofluorescence

ROS assay kit (S0033S, Beyotime, Shanghai, China) was used to detect the intracellular ROS level. The lipid peroxidation level was detected using a C11 BODIPY 581/591 fluorescent probe (D3861, Thermofisher, USA). RhoNox-1, a fluorescent probe for the specific detection of divalent iron ions (HY-D1533, MedChemExpress, Shanghai, China) was used to detect the Fe^2+^ level. All operations were performed according to the manufacturer’s instructions.

The RPE cells underwent fixation using 4% paraformaldehyde (PFA) for 10 min. They were then treated with 0.5% Triton-X-100 for 10 min. Following this, the cells were blocked using a solution of 5% BSA in PBS for 2 h at room temperature (RT). The primary antibodies against CYP4V2 (diluted 1:200, ab69392, Abcam, USA) were applied and allowed to incubate overnight at 4 °C. The secondary antibodies were subsequently applied and incubated at RT for 1 h. DAPI (62,248, Thermofisher, USA) was used to stain the cell nuclei. The imaging was performed using an Olympus FV3000 microscope (Olympus, Tokyo, Japan).

### Southern blotting & Western blotting

Genomic DNA was extracted from the mice’s tail tips and amplified with One Step Mice Genotyping Kit (PD101, Vazyme, Jiangsu, China) according to the manufacturer’s instructions. The primers for amplifying were: F, TGCAGAAAATGTGGAGGTAATTTT; R, AGAAGTCCTAGGCCAAGCCA. Western blot analysis was performed according to the standard procedure to analyze the expression of FTH1 (1:1000, A19544, Abclonal, China), NCOA4 (1:1000, ab314553, Abcam, USA), β-actin (1:5000, 8457, CST, USA), ACSL4 (1:1000, A20414, Abclonal, China), GPX4 (1:1000, Protein Tech, USA).

### Real-time PCR

Transcript levels of FTL (F, CCTACCTCTCTCTGGGCTTCT; R, CCACGCTGGTTTTGCATCTT), FTH1 (F, GGTGCGCCAGAACTACCAC; R, TCGCGGTCAAAGTAGTAAGACATGG), NCOA4 (F, CACTCGGACCTGGAGCAG; R, CCTCCGTGCATCACTACACC), GPX4 (F, GAAGATCCAACCCAAGGGCA; R, GACGGTGTCCAAACTTGGTG) in *CYP4V2* mutated RPE were analyzed by RT-PCR. Total RNA was extracted using Trizol reagent and quantified spectrophotometrically at 260 nm. Realtime PCR was performed using Biorad CFX96 (Biorad, USA). The qPCR protocol consisted of initial denaturation at 95 °C for 30 s,40 cycles of amplification at 95 °C for 5 s, 60 °C for 30 s, and a final melting curve stage. mRNA levels were calculated using the 2^−ΔΔCT^ method and normalized to ACTIN (F, TCACCAACTGGGACGACAT; R, ATCTGGGTCATCTTCTCGC) levels.

### Statistical analysis

The statistical analysis of all data was conducted using version 9.5.0 of the GraphPad Prism software package. For independent datasets with a Gaussian distribution, a two-tailed t-test was used for two-way comparisons. To analyze statistically significant differences between two or more groups, a one-way ANOVA with a post hoc Scheffe test was performed. The null hypothesis was rejected based on a p-value < 0.05. Standard deviation (SD) was represented by error bars in all graphical representations.

## Results

### Progressive degeneration and lipid accumulation of retina in *Cyp4v3* KO mice

**Fig. 1:**
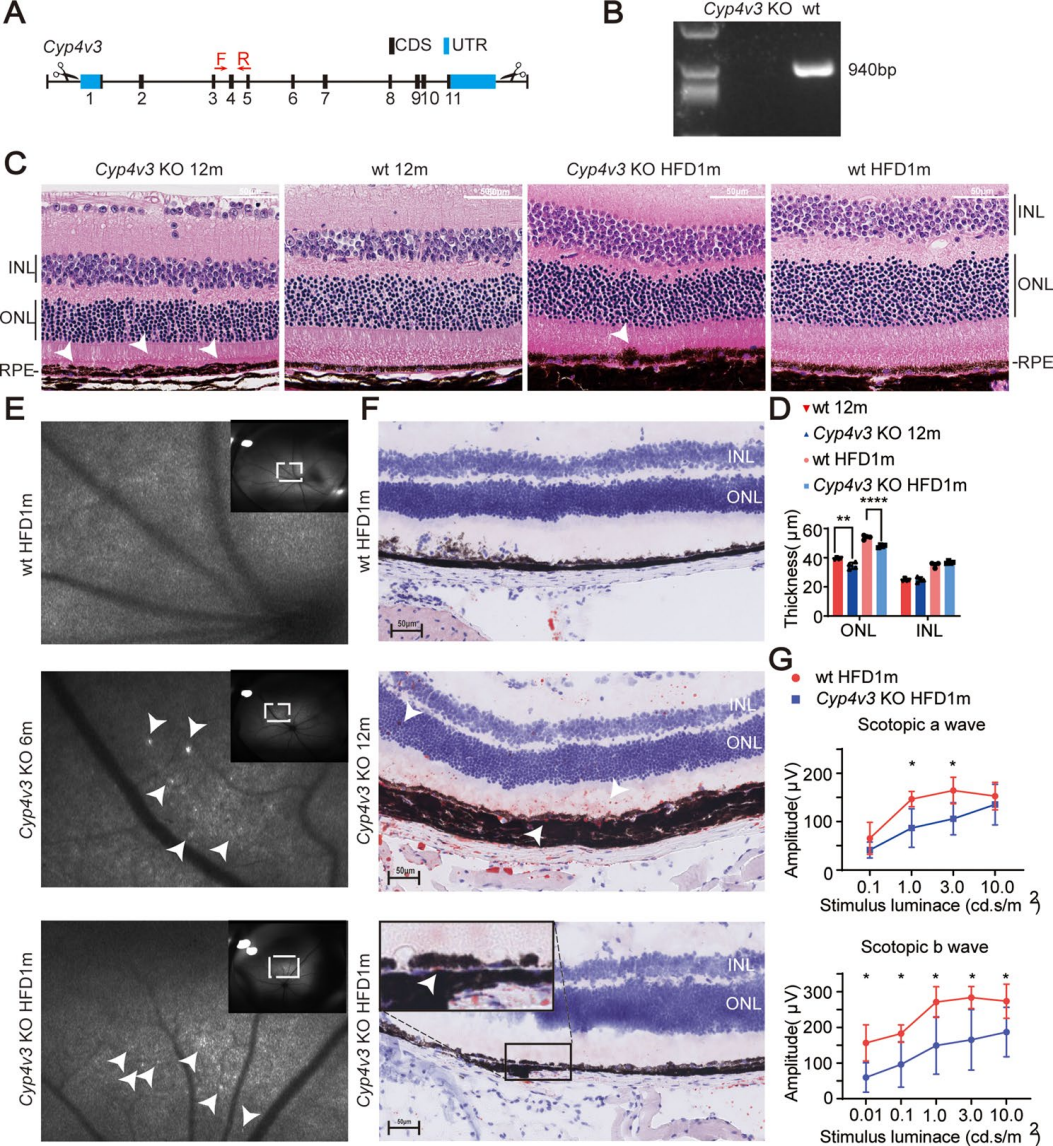
Progressive degeneration and lipid accumulation of retina in *Cyp4v3* KO mice. **(A)** Strategy for construction of *Cyp4v3* KO mice, the scissors indicated sgRNAs, which deleted the entire gene. Red arrows indicated primers for southern blotting. **(B)** Southern blotting showed a 940 bp band in wt mice, and a blank band in *Cyp4v3* KO mice. **(C)** HE images of 12-month-old wt mice (wt 12 m), 12-month-old *Cyp4v3* KO mice (*Cyp4v3* KO 12 m), wt mice fed with 1-month HFD (wt HFD1m, 7-week-old at experiment), *Cyp4v3* KO mice fed with 1-month HFD (*Cyp4v3* KO HFD1m, 7-week-old at experiment). White arrows in *Cyp4v3* KO 12 m mice and *Cyp4v3* KO HFD1m mice showed RPE hyperplasia and disorganization in focal areas. **(D)** ONL and INL thickness of wt 12 m, *Cyp4v3* KO 12 m, wt HFD1m, *Cyp4v3* KO HFD1m (*n* = 4). **(E)** UWF-AF of wt mice with wt HFD 1 m, 6-month-old *Cyp4v3* KO mice (*Cyp4v3* KO 6 m), *Cyp4v3* KO HFD 1 m. White arrows point out crystalline deposits on the fundus. **(F)** Oil red staining of *Cyp4v3* KO 12 m, wt HFD1m, *Cyp4v3* KO HFD1m, red deposits dispersed throughout the retina of *Cyp4v3* KO 12 m, red deposits were found beneath RPE in *Cyp4v3* KO HFD1m. **(G)** Amplitude of scotopic a-wave and b-wave by ERG in wt HFD1m and *Cyp4v3* KO HFD1m (*n* = 5). KO, knock out; wt, wild-type; HE, hematoxylin eosin stain; HFD, high-fat diet; ONL, out nuclear layer; INL, inner nuclear layer; RPE, retinal pigment epithelium; UWF-AF, ultra-wide field fundus autofluorescence. *P < 0.05. **P < 0.01, ****P < 0.0001

CRISPR-Cas9 technology was used to create *Cyp4v3* KO mice based on the strategy employed in a previous study [11]. This involved the complete deletion of the mice *Cyp4v3* locus, which is the homologous gene to human *CYP4V2* (Fig. 1. A). The genotype of *Cyp4v3* KO mice was verified by Southern blotting (Fig. 1. B). Furthermore, different ages of *Cyp4v3* KO mice exhibited retinal lesions and functional changes. UWF-AF imaging of 6-month-old *Cyp4v3* KO mice displayed crystalline deposits on the fundus which are characteristic features observed in BCD patients (Fig. 1. E). HE staining revealed decreased thickness of the outer nuclear layer (ONL) and RPE hyperplasia and disorganization in focal areas of 12-month-old *Cyp4v3* KO mice (Fig. 1.C&D). Oil Red O staining revealed the presence of lipid droplets dispersed throughout the inner nuclear layer (INL), ONL, and sub-RPE layer in the retina of 12-month-old *Cyp4v3* KO mice. Notably, the majority of these lipid droplets were found to accumulate specifically in the sub-RPE layer (Fig. 1. F).

By feeding *Cyp4v3* KO mice with an HFD diet, the phenotype can be accelerated. Retina function assessed by ERG revealed deficiency in *Cyp4v3* KO mice with an HFD diet for only 1 month. The amplitude of scotopic a-wave and b-wave was reduced compared to WT mice with the same HFD diet (Fig. 1. G). Crystalline deposits were detected on UWF-AF fundus imaging of *Cyp4v3* KO mice with a 1-month HFD diet (Fig. 1. E). HE staining and Oil Red O staining also demonstrated a similar phenotype in *Cyp4v3* KO mice after 1 month of HFD, resembling that observed in 12-month-old *Cyp4v3* KO mice (Fig. 1.C&D&F).

Inflammatory responses were observed in both 12-month-old *Cyp4v3* KO mice and *Cyp4v3* KO mice with a 1-month HFD diet, as evidenced by IBA1 staining which indicated activated microglia cells (Supplement Fig. 1). All the above phenotypes indicated *Cyp4v3* KO mice can mimic human BCD phenotype to some extent. Furthermore, these results indicate the need for a more detailed investigation into lipid metabolism in this model.

### Abnormal lipid metabolism and ferroptosis in RPE of *Cyp4v3* KO mice

**Fig. 2:**
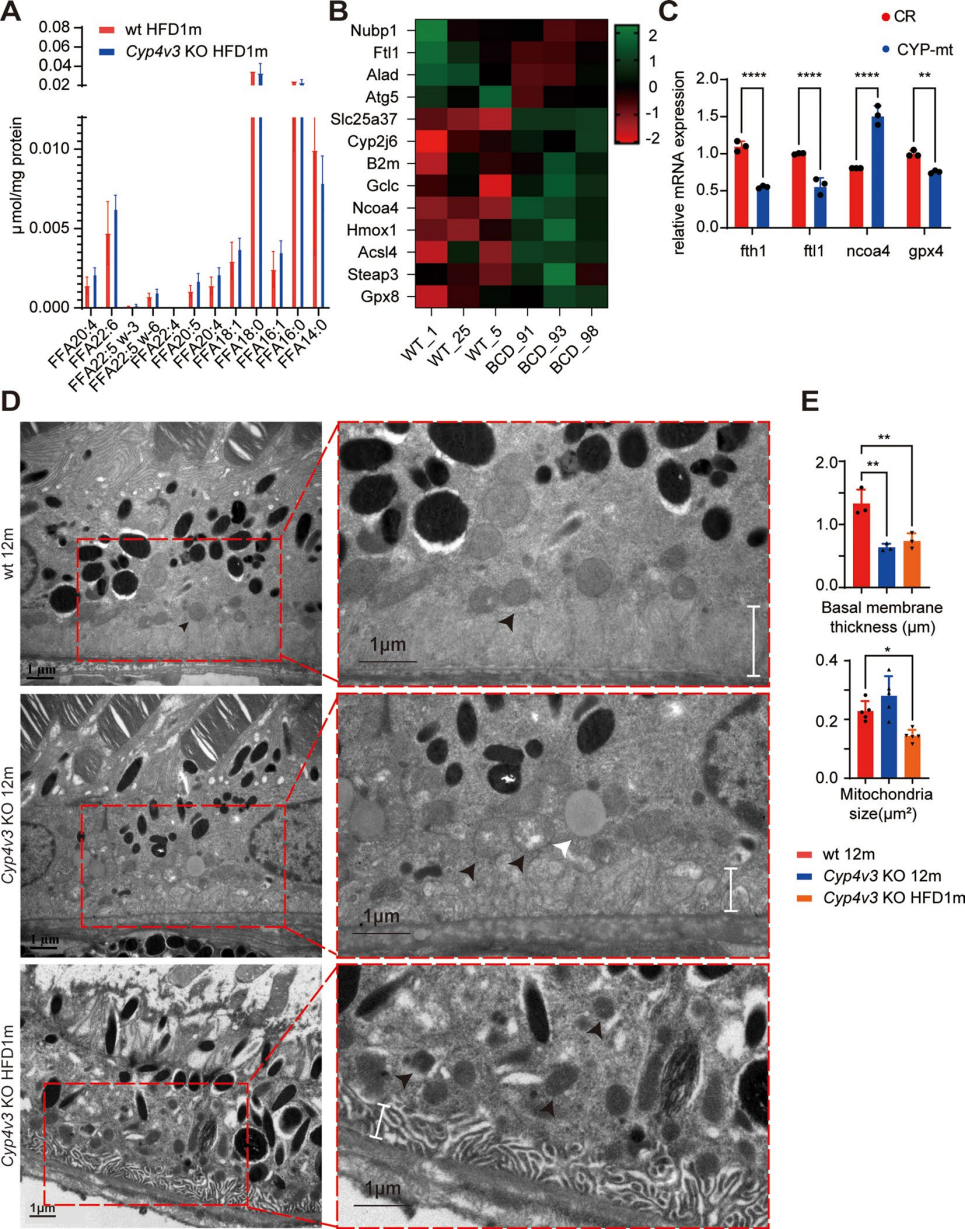
Abnormal lipid metabolism and ferroptosis in RPE of Cyp4v3 KO mice. **(A)** FFA profiling of *Cyp4v3* KO HFD1m and wt HFD1m (*n* = 3), EPA (C20:5), AA (C20:4), DHA (C22:6), DPA (C22:6) showed upward trends in RPE of *Cyp4v3* KO HFD1m. **(B)** Transcriptome profiling of *Cyp4v3* KO HFD1m and wt HFD1m mice, significant changes of iron homeostasis genes were revealed (*n* = 3). **(C)** mRNA expression level of differential genes in *CYP4V2* mutated RPE cell-line (CYP-mt) and control RPE cell-line (CR) (*n* = 3). **D&E.** TEM of RPE in wt 12 m, *Cyp4v3* KO 12 m, and *Cyp4v3* KO HFD1m. Ragged mitochondrial (black arrows) and lipid droplets (white arrows) have been detected in RPE of *Cyp4v3* KO 12 m mice, while decreased size of mitochondrial have been detected in RPE of *Cyp4v3* KO HFD 1 m mice (black arrows, *n* = 5). The thickness of the RPE basal membrane decreased in both *Cyp4v3* KO 12 m mice and *Cyp4v3* KO HFD 1 m mice (white lines, *n* = 3). FFA, free fatty acid; KO, knock out; wt, wild-type; HFD, high-fat diet; EPA, eicosapentaenoic acid; AA, arachidonic acid; DHA, docosahexaenoic Acid; DPA, docosapentaenoic acid; mt, mutated; CR, control; RPE, retinal pigment epithelium; TEM, transmission electron microscope; The full names of genes in Fig. 2.B were shown in abbreviation index. *P < 0.05, **P < 0.01, ***P < 0.001

To further investigate the abnormal lipid metabolism in *Cyp4v3* KO mice retina, FFA profiling of RPE was performed on *Cyp4v3* KO HFD 1 m mice and WT HFD 1 m mice. No significant differences in FFAs were detected between the two groups. Several PUFAs including EPA (C20:5), AA (C20:4), DHA (C22:6), and DPA (C22:6) showed upward trends in *Cyp4v3* KO mice (Fig. 2. A). Overloaded PUFAs, which provide the substrate for lipid peroxidation, have been reported to be a crucial factor in ferroptosis.

To verify whether ferroptosis is involved in the pathogenesis of BCD, we performed TEM and IF staining to measure biochemical indicators of ferroptosis. TEM of retinal historical sections revealed mitochondrial abnormalities in the RPE of *Cyp4v3* KO 12 m mice and *Cyp4v3* KO HFD 1 m mice. Ragged mitochondrial and lipid droplets have been detected in RPE of *Cyp4v3* KO 12 m mice (Fig. 2. D), while decreased size of mitochondrial have been detected in RPE of *Cyp4v3* KO HFD 1 m mice (Fig. 2. D&E). The thickness of the RPE basal membrane decreased in both *Cyp4v3* KO 12 m mice and *Cyp4v3* KO HFD 1 m mice (Fig. 2. D&E).

**Fig. 3:**
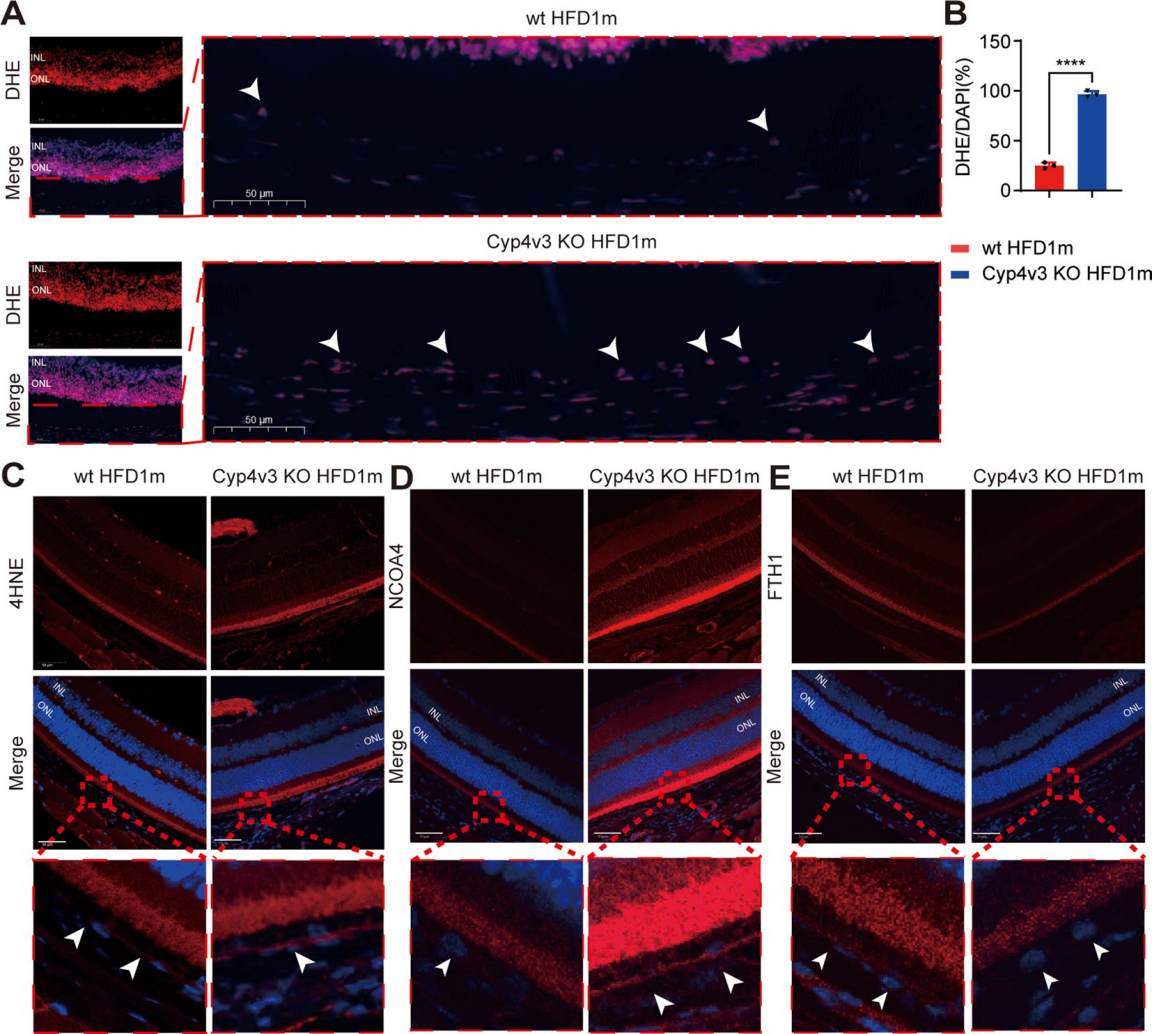
Abnormal lipid metabolism and ferroptosis in the RPE of *Cyp4v3* KO mice. **A&B.** DHE staining of *Cyp4v3* KO HFD1m and wt HFD1m mice, ROS level of RPE increased significantly in *Cyp4v3* KO HFD1m mice (white arrows, *n* = 3).**C&D&E.** 4-HNE, NCOA4 and FTH1 staining of *Cyp4v3* KO HFD1m and wt HFD1m mice, RPE was amplified in red blocks. The 4-HNE staining revealed increased levels of lipid peroxidation in the RPE of *Cyp4v3* KO HFD1m mice, along with elevated levels of NCOA4. Additionally, the FTH1 level was found to be decreased in *Cyp4v3* KO mice. KO, knock out; wt, wild-type; DHE, dihydroethidium; 4-HNE, 4-Hydroxynonenal; NCOA4, nuclear receptor coactivator 4; FTH1, ferritin heavy chain 1. ****P < 0.0001

Furthermore, DHE and 4-HNE staining has been used to detect ROS and lipid peroxidation levels in the retina of *Cyp4v3* KO HFD 1 m mice. ROS level of RPE increased significantly in *Cyp4v3* KO HFD 1 m mice (Fig. 3. A&B), as well as the increased level of lipid peroxidation (Fig. 3. C). All the above results indicated ferroptosis was involved in the RPE of *Cyp4v3* KO mice.

To further explore the specific mechanism of RPE ferroptosis in *Cyp4v3* KO mice, transcriptome profiling and analysis of RPE have been performed in *Cyp4v3* KO HFD 1 m mice and WT HFD 1 m mice. Transcriptome analysis revealed significant changes of iron homeostasis genes in the transcriptome level of *Cyp4v3* KO HFD 1 m mice compared to WT mice (Fig. 2. B) and was confirmed by RT-PCR in *CYP4V2* mutated RPE (Fig. 2. C). Among these genes, upregulation of ACSL4 and NCOA4 was detected. ACSL4 is a key enzyme that activates PUFAs to facilitate ferroptosis, while NCOA4 is a receptor of ferritinopahgy, which plays a crucial role in the iron homeostasis of several tissues as well as the retina. NCOA4 IF of the retinal section showed increased expression in 1 m HFD *Cyp4v3* KO mice (Fig. 3. D). The NCOA4 binding protein, FTH1, decreased slightly in the RPE of 1 m HFD *Cyp4v3* KO mice (Fig. 3. E).

### Abnormal ferrous iron accumulation and ferroptosis in *CYP4V2* mutated RPE

**Fig. 4:**
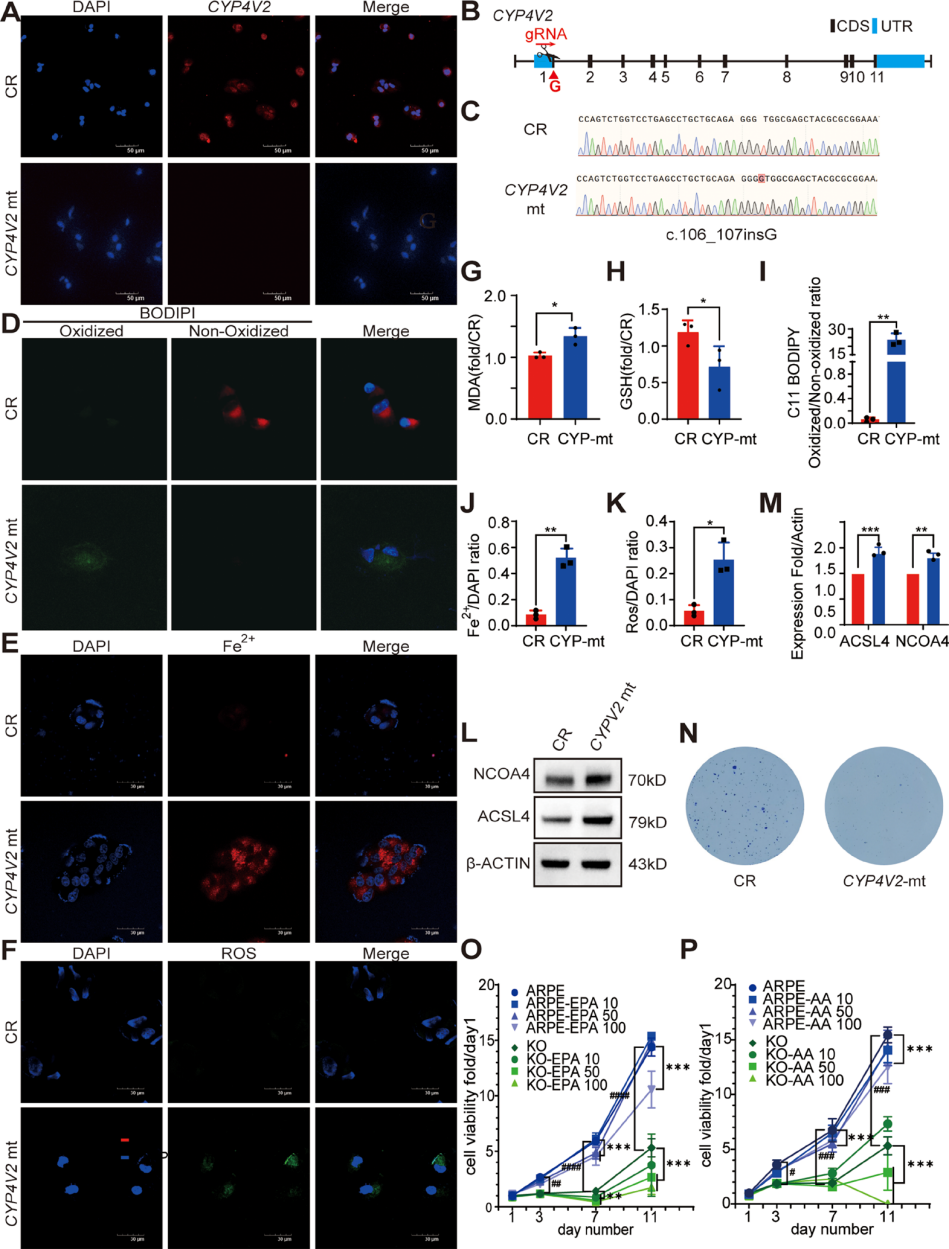
Abnormal ferrous iron accumulation and ferroptosis in *CYP4V2* mutated RPE. **(A)** Immunofluorescence staining of *CYP4V2* in *CYP4V2* mutated (mt) RPE and control (CR) RPE. **(B)** Strategy for construction of *CYP4V2* mt RPE, the scissor indicated sgRNA, which located at 70 bp after initiation codon. **(C)** Sanger sequencing results of *CYP4V2* mt RPE and CR cells, there was 1 bp insertion at c.106 (red block). **D&I.** C11 BODIPY staining showed increased lipid peroxidation levels in *CYP4V2* mt RPE compared to CR RPE (*n* = 3). **E&J.** RhoNox-1 staining showed increased ferrous iron level in *CYP4V2* mt RPE compared to CR RPE (*n* = 3). **F&K.** Increased ROS level in *CYP4V2* mt RPE compared to CR RPE (*n* = 3). **G.** MDA level of *CYP4V2* mt RPE and CR RPE (*n* = 3). **H.** GSH level of *CYP4V2* mt RPE and CR RPE (*n* = 3). **L&M.** Increased NCOA4 and ACSL4 level in *CYP4V2* mt RPE compared to CR RPE by WB (*n* = 3). For Fig 4. G-M, *P<0.05, **P<0.01, ***P<0.001. **N.** Clone formation revealed impaired cell proliferation in *CYP4V2* mt RPE compared to CR RPE. **O&P.** EPA and AA treatment defected cell proliferation in *CYP4V2* mt RPE and CR RPE. Cell proliferation rate decreased significantly in *CYP4V2* mt RPE compared to control without PUFAs administration from day 3 to day 11 (*n* = 3). ##*P* < 0.01, ###*P* < 0.001, ####*P* < 0.0001. At day 7, 50µM and 100µM EPA affected cell viability both in *CYP4V2* mt RPE and CR RPE, this affection lasted in *CYP4V2* mt RPE to day 11, while in CR RPE, only 100µM EPA affected cell viability on day 11. For AA, at day 7, 100µM AA affected cell viability in control cells, this affection lasted until day 11; in *CYP4V2* mt RPE, 50µM and 100µM AA affected cell viability severely at day 11. **P < 0.01, ***P < 0.001,****P < 0.0001. Mt, mutated; CR, control; MDA, malondialdehyde; GSH, glutathione; ROS, reactive oxygen species; NCOA4, nuclear receptor coactivator 4; ACSL4, acylcoenzyme A (CoA) synthetase long-chain family member 4; WB, western blotting; EPA, eicosapentaenoic acid; AA, arachidonic acid

To further investigate the pathogenesis of BCD in virtro, *CYP4V2* mt RPE has been generated by CRISPR-Cas9 technology. The SgRNA target site was designed to be located at 70 bp after the initiation codon (Fig. 4. B), which led to a 1-bp insertion at c.106. This insertion caused a mistranslation of an amino acid after amino 37 and terminated after amino 43. The genotype of *CYP4V2* RPE is c.106_107insG, V37Gfs*43. Sequencing verification had been confirmed before following experiments (Fig. 4. C). CYP4V2 expression was confirmed by IF, which showed rarely CYP4V2 expression in *CYP4V2* mt cells (Fig. 4. A).

**Fig. 5:**
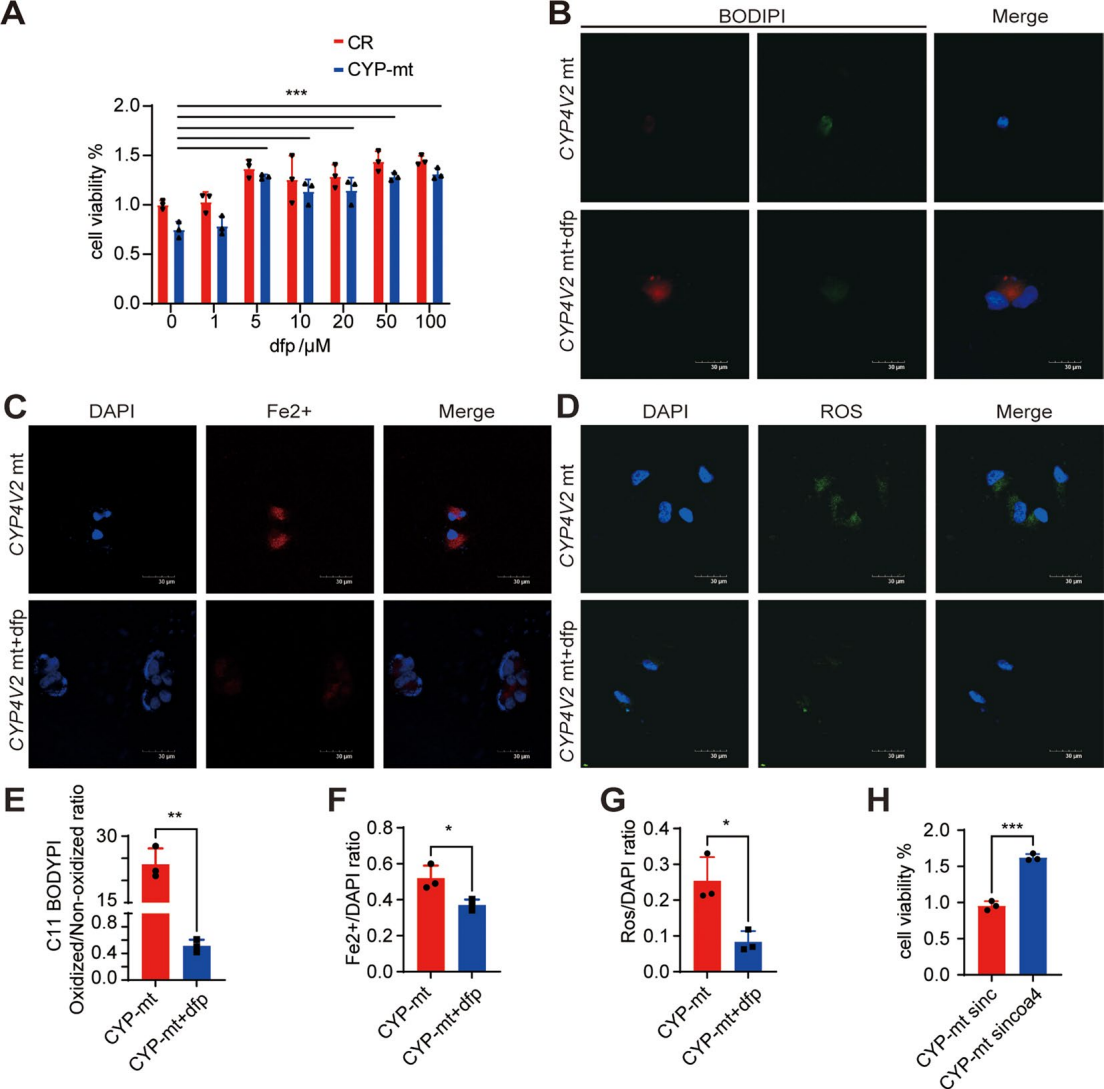
DFP alleviated ferroptosis in CYP4V2 mt RPE. **A.** DFP increased cell viability in a dose-dependent manner in *CYP4V2* mt RPE. **B&E.** Decreased lipid peroxidation level in *CYP4V2* mt RPE with 50µM DFP pre-treatment (n = 3). **C&F.** Decreased ferrous iron level in CYP4V2 mt RPE with 50µM DFP pre-treatment (*n* = 3). **D&G.** Decreased ROS level in CYP4V2 mt RPE with 50µM DFP pre-treatment (*n* = 3). **H.** Increased cell viability in *CYP4V2* mt RPE with siNCOA4 (*n* = 3). DFP, deferiprone; Mt, mutated; CR, control; ROS, reactive oxygen species. **P < 0.01, ***P < 0.001,****P < 0.0001

Lipid oxidation biomarkers including MDA and GSH levels were tested in both *CYP4V2* mt RPE and WT RPE and showed increased MDA levels and decreased GSH levels in *CYP4V2* mt RPE. (Fig. 4. G&H). IF staining revealed increased levels of lipid peroxidation (Fig. 4. D&I), ROS (Fig. 3. F&K), and ferrous iron accumulation (Fig. 4. E&J) in *CYP4V2* mt RPE. Furthermore, Western blotting revealed increased ACSL4 and NCOA4 levels in *CYP4V2* mt RPE (Fig. 4. L&M). Clone formation revealed impaired cell proliferation in *CYP4V2* mt RPE (Fig. 5. N).

To verify whether PUFAs accumulation facilitated RPE cell death, 11 days of PUFAs exposure including AA and EPA was performed on *CYP4V2* mt RPE and control RPE (Fig. 5. O&P). Cell proliferation rate decreased significantly in *CYP4V2* mt RPE compared to control without PUFAs administration from day 3 to day 11. At day 7, 50µM and 100µM EPA affected cell viability both in *CYP4V2* mt RPE and control cells, this affection lasted in *CYP4V2* mt RPE to day11, while in control RPE, only 100µM EPA affected cell viability on day11. For AA, at day 7, 100µM AA affected cell viability in control cells, this affection lasted until day 11; in *CYP4V2* mt RPE, 50µM and 100µM AA affected cell viability severely at day 11. These indicated EPA and AA accumulation play a vital role in RPE cell death both in healthy RPE and *CYP4V2* mt RPE.

### DFP alleviated ferroptosis in *CYP4V2* mt RPE

DFP is an oral iron chelator that can be used to alleviate iron-overloaded diseases efficiently [28]. We used DFP in vitro to test its effect of inhibiting ferroptosis in *CYP4V2* mt RPE. In *CYP4V2* mt RPE, DFP increased cell viability in a dose-dependent manner (Fig. 5. A), which revealed that 50µM DFP pre-treatment can increase *CYP4V2* mt RPE viability efficiently. Furthermore, with 50µM DFP pre-treatment, ROS, C11 BODIPY, and FeOrange staining were used to examine the effect of inhibiting ferroptosis on *CYP4V2* mt RPE, which revealed a significant reduction of iron accumulation (Fig. 5. C&F). Lipid peroxidation level and ROS level have also decreased by DFP treatment (Fig. 5. B&E, D&G). Inhibiting NCOA4 expression by siRNA also resulted in enhanced cell viability in *CYP4V2* mt RPE (Fig. 5. H).

### DFP ameliorated retinal defection in *Cyp4v3*KO mice

**Fig. 6:**
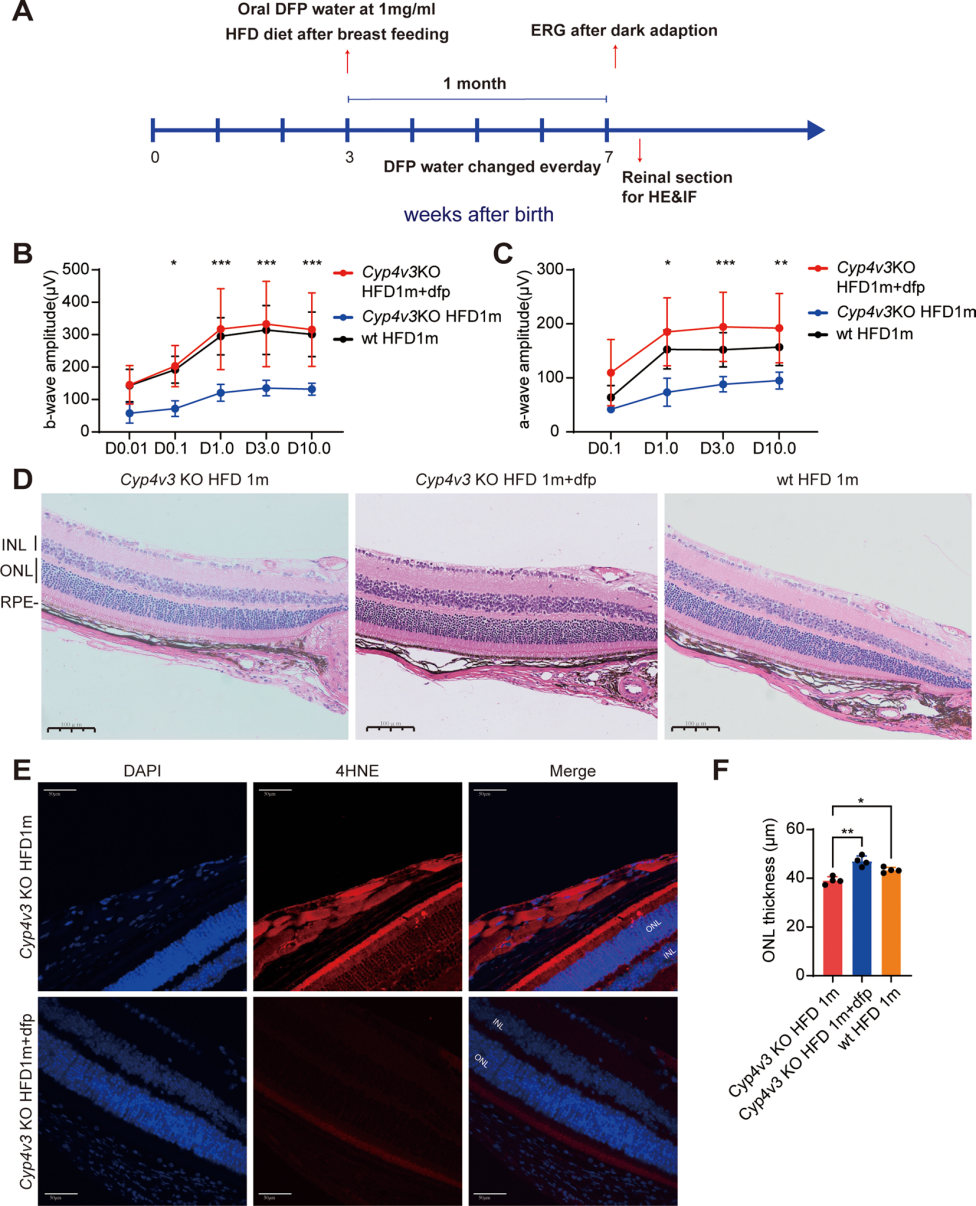
DFP ameliorated retinal defection in Cyp4v3 KO mice. **A.** Timeline of *Cyp4v3* KO mice with HFD and DFP treatment. Mice with HFD were fed after breastfeeding and were 7 weeks old at the experiment. **B&C.** Amplitude of scotopic a-wave and b-wave by ERG in *Cyp4v3* KO HFD1m mice with DFP compared to *Cyp4v3* KO HFD 1 m mice (*n* = 5) which showed notable improvements in retinal function in *Cyp4v3* KO HFD1m mice with DFP. **D&F.** HE staining of *Cyp4v3* KO HFD1m mice with DFP and *Cyp4v3* KO HFD 1 m mice (*n* = 4) which showed increased thickness of ONL in *Cyp4v3* KO HFD1m mice with DFP. **E.** 4-HNE staining of *Cyp4v3* KO HFD1m mice with DFP and *Cyp4v3* KO HFD 1 m mice which showed decreased lipid peroxidation level in *Cyp4v3* KO HFD1m mice with DFP. DFP, deferiprone; KO, knock out; HFD, high-fat diet; ERG, electroretinogram; HE, hematoxylin and eosin; ONL, out nuclear layer; 4-HNE, 4-Hydroxynonenal. *P < 0.05, **P < 0.01, ***P < 0.001,****P < 0.0001

To assess the efficacy of DFP in an in vivo model, oral DFP treatment was performed concurrently with the HFD diet (Fig. 6. A). As an oral iron chelator, DFP is proven to be able to pass through the retina-blood barrier[19]. Oral administration of DFP in *Cyp4v3* KO mice yielded notable improvements in retinal function examined by ERG (Fig. 6. B&C). HE staining revealed evident morphological recovery characterized by thickening ONL (Fig. 6. D&F). Furthermore, 4-HNE staining demonstrated a reduction in lipid peroxidation levels of the retina especially the RPE layer of *Cyp4v3* KO mice after DFP treatment (Fig. 6. E). Collectively, these findings underscore the potential of DFP to mitigate ferroptosis and restore retinal function in *Cyp4v3* KO mice.

## Discussion

As a progressive retina degeneration disease, there is currently no clinical therapy approach available for BCD patients. Although several studies have reported gene replacement therapy using AAV-hCYP4V2 in mice models [11, 29], or patients-derived iPSC-RPE [13, 28], offering renewed hope for future treatment, no clinical trial results have been reported to date. Therefore, exploring the pathogenesis of the disease is crucial for improved treatment. Several researchers have developed models to mimic the BCD phenotype [11–14, 27], leading to the consensus that abnormal lipid metabolism is involved in the pathogenesis of BCD. However, the precise mechanism by which lipid metabolism impacts RPE health in BCD remains unclear. In this research, the mechanism of RPE cell death in BCD was explored: PUFA accumulation and ferroptosis were detected in the RPE of *Cyp4v3* KO mice model. Ferroptosis and abnormal iron homeostasis were detected in the *CYP4V2* mt RPE model and were facilitated by AA or EPA administration. Furthermore, DFP, an oral iron chelator, alleviated the phenotype both in vitro and in vivo.

PUFAs play critical roles in biogenesis as structural components of cellular membranes [30]. Photoreceptor outer segments, crucial components of the retina, consist of unique stacked disks comprised of cellular membranes. This distinct structure leads to a substantial increase in PUFA content in the retina [31]. RPE, located adjacent to photoreceptors, supports their function by phagocytizing shed photoreceptor OS a tenth of daily. Thus, PUFA metabolism in RPE is highly active [32]. Among the eye, *CYP4V2* is found to be highly expressed in the RPE and is involved in lipid metabolism, especially in hydroxylating medium-long chain PUFA [7]. Our study (Fig. 2. A) and previous research [9–14] have confirmed abnormal PUFA accumulation resulting from mutated *CYP4V2*.

In this study, enzymatic digestion was used to isolate RPE, resulting in a limited quantity of RPE samples and a low sample size, thus leading to statistically insignificant differences. However, we observed an upward trend in EPA (C20:5), AA (C20:4), DHA (C22:6), and DPA (C22:6) levels in the RPE of *Cyp4v3* KO mice. Different PUFA administrations including EPA (omega-3 PUFA) and AA (omega-6 PUFA) were then used, which affected cell viability at low concentrations (50µM) in *CYP4V2* mt RPE (Fig. 4. O&P), indicating that the accumulation of PUFAs in RPE contributes to the pathogenesis of BCD.

Dietary PUFAs act as beneficial nutrient supplements in several diseases, such as cancers, cardiovascular diseases, mental disorders as well and age-related macular degeneration (AMD) [32–36]. While PUFAs played dual roles in retinal physiology [37]. It is reported that high blood omega-6 PUFAs specifically AA increased the odds ratio of having neovascular AMD [38]. In BCD patient-derived iPSC-RPE, EPA and AA treatment increased mitochondrial reactive oxygen species and impaired mitochondrial respiratory functions [13].

Ferroptosis is defined as a specific type of cell death that depends on iron-dependent lipid peroxidation. Ferrous iron (Fe^2+^) instigates the generation of hydroxyl radicals, such as ROS, via the Fenton reaction [15]. PUFAs, which can be incorporated into membrane lipids, act as the essential substrate of lipid oxidation [22]. When lipid peroxidation exceeds the capacity of the cellular antioxidant defense system, including the GSH system, ferroptosis ensues [39]. In this study, we observed evidence of ferroptosis both in vitro and in vivo of BCD. By DHE and 4-HNE staining, lipid peroxidation was confirmed in the RPE of *Cyp4v3* KO mice (Fig. 3. A&C). Similarly, in *CYP4V2* mt RPE, lipid peroxidation was evident through ROS and C11 BODIPY staining (Fig. 4. D&I, F&K), as well as quantification of MDA level (Fig. 4. G). Furthermore, GSH level was found to be reduced in *CYP4V2* mt RPE (Fig. 4. H). TEM revealed mitochondria defection in the RPE of *Cyp4v3* KO mice (Fig. 2. D&E). Excessive ACSL4 expression, an enzyme that facilitates the integration of overloaded PUFA into membrane lipid and contributes to ferroptosis, was identified in both *CYP4V2* mt RPE (Fig. 4. L&M) and RPE of *Cyp4v3* KO mice (Fig. 2. B). Importantly, all observed phenotypes could be alleviated by the treatment of iron chelator, DFP (Figs. 5 and 6).

In *CYP4V2* mt RPE, excess ferrous iron was detected by ferrous iron staining (Fig. 4. E&J). Levels of iron storage protein, light ferritin chains (FTL1), and heavy ferritin chains (FTH1) [40], were found to be decreased in RPE of *Cyp4v3* KO mice (Fig. 2.B&K), leading to an increase of ferrous iron in the RPE. Transcriptome profiling revealed the upregulation of the nuclear receptor co-activator 4 (NCOA4) (Fig. 2. B&C), which was confirmed both in vitro and in vivo (Fig. 3. D, Fig. 4. L&M). NCOA4 functions as a ferritinophagy regulator, binding with ferritin and facilitating the degradation of ferritin into ferrous iron [41]. Inhibiting NCOA4 expression by siRNA resulted in enhanced cell viability in *CYP4V2*-mt RPE (Fig. 5. H). Additional studies are needed to explore the changes in iron autophagy and iron homeostasis by *CYP4V2* mutations.

In summary, this study has first revealed that ferroptosis is mainly involved in the pathogenesis of BCD which caused by the mutations of *CYP4V2*. Mutations in *CYP4V2* resulted in the accumulation of PUFAs, especially in the RPE. The upregulation of ACSL4 promotes the integration of PUFAs into membrane lipids, serving as substrates for lipid peroxidation. Furthermore, the upregulation of NCOA4 in BCD indicates that NCOA4-mediated ferritinophagy plays a role in the excess accumulation of ferrous iron. The oxidative stress generated by the Fenton reaction overwhelms the redox system, leading to ferroptosis. The oral chelator DFP has been investigated for its potential to reverse the ferroptosis phenotype. These findings indicate that targeting ferroptosis could be a promising approach for treating BCD, complementing gene therapy efforts.

### Electronic supplementary material

Below is the link to the electronic supplementary material.

Supplementary Material 1

## Data Availability

No datasets were generated or analysed during the current study.

## Abbreviations

BCD: Bietti Crystalline Dystrophy
PUFA: Polyunsaturated fatty acid
CYP4V2: Cytochrome P450 (CYP) family 4 subfamily V
Cyp4v3: Cytochrome P450, family 4, subfamily v, polypeptide 3
KO: Knock out
RPE: Retinal pigment epithelium
ROS: Reactive oxygen species
DFP: Deferiprone
HFD: High-fat diet
EPA: Eicosapentaenoic acid
AA: Arachidonic acid
NCOA4: Nuclear receptor coactivator 4
DHA: Docosahexaenoic acid
AMD: Age-related macular degeneration
ACSL4: Acylcoenzyme A (CoA) synthetase long-chain family member 4
ARPE-19: Adult RPE cell line-19
DMEM: Dulbecco’s modified Eagle’s medium
FBS: Fetal bovine serum
UWF-AF: Ultra-wide field fundus autofluorescence
ERG: Electroretinogram
HE: Hematoxylin and eosin
4-HNE: 4-Hydroxynonenal
FTH1: Ferritin heavy chain 1
IBA1: Induction of brown adipocytes 1
TEM: Transmission electron microscopy
MDA: Malondialdehyde
GSH: Glutathione
PFA: Paraformaldehyde
RT: Room temperature
DAPI: Diamidino-phenyl-indole
GPX4: Glutathione peroxidase 4
FFA: Free fatty acid
ONL: Outer nuclear layer
INL: Inner nuclear layer
Nubp1: NUBP iron-sulfur cluster assembly factor 1
Ftl1: Ferritin light polypeptide 1
Alad: Aminolevulinate dehydratase
Atg5: Autophagy related 5
Slc25a37: Solute carrier family 25 member 37
Cyp2j6: Cytochrome P450, family 2, subfamily j, polypeptide 6
B2m: Beta-2-microglobulin
Gclc: Glutamate-cysteine ligase catalytic subunit
Hmox1: Heme oxygenase 1
Steap3: STEAP family member 3
Gpx8: Glutathione peroxidase 8
Mt: Mutated
CR: Control
DHE: Dihydroethidium
