# Supplementary material for: Uncovering the role of ferroptosis in Bietti crystalline dystrophy and potential therapeutic strategies
Source: Cell Commun Signal. 2024 Jul 11;22:359. doi: 10.1186/s12964-024-01710-x (PMC11241923; doi:10.1186/s12964-024-01710-x)
Supplement: Supplementary file 1 — Supplementary Material 1 [file 12964_2024_1710_MOESM1_ESM.docx]

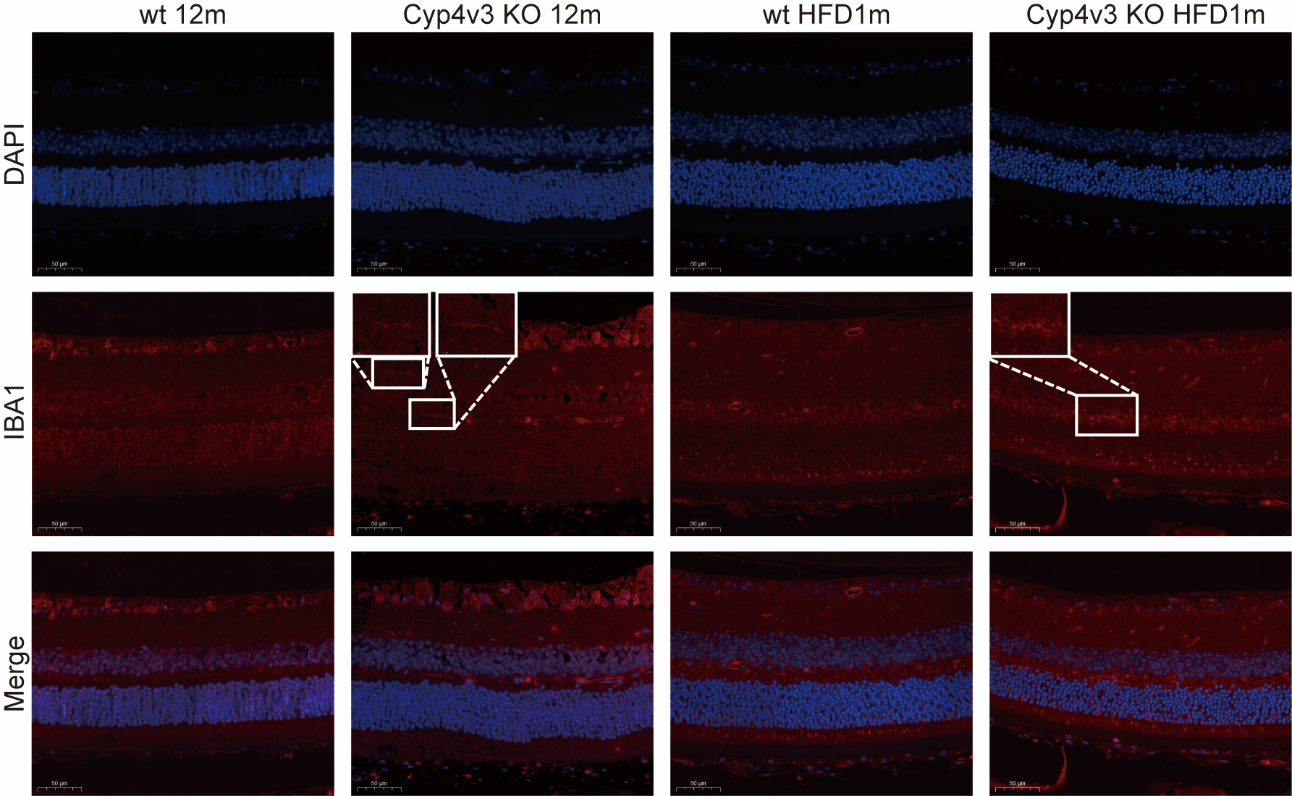


**Supplement Figure 1.** Iba1 staining of wt 12m, *Cyp4v3* KO 12m, wt HFD 1m, *Cyp4v3* KO HFD 1m mice, white blocks pointed out star-like activated microglia cells indicated by IBA1. IBA1, induction of brown adipocytes 1; KO, knock out; wt, wild type; HFD, high-fat diet.
